# Instruments to measure environmental and personal radiofrequency-electromagnetic field exposures: an update

**DOI:** 10.1007/s13246-022-01146-y

**Published:** 2022-06-23

**Authors:** Chhavi Raj Bhatt, Stuart Henderson, Chris Brzozek, Geza Benke

**Affiliations:** 1 Radiation Research and Advice Section, Radiation Health Services Branch, Australian Radiation Protection and Nuclear Safety Agency, 619 Lower Plenty Rd, Yallambie, VIC 3085 Australia; 2 Monash Centre for Occupational and Environmental Health, School of Public Health and Preventive, Medicine, Monash University, 553 St Kilda Rd, Melbourne, VIC 3004 Australia

**Keywords:** Exposimeters, Exposure assessment, Mobile phone exposures, Monitoring systems, Radiofrequency-electromagnetic exposures, Radiofrequency-electromagnetic exposure assessment

## Abstract

Modern human populations are exposed to anthropogenic sources of radiofrequency-electromagnetic fields (RF-EMFs), primarily to telecommunication and broadcasting technologies. As a result, ongoing concerns from some members of the public have arisen regarding potential health effects following RF-EMF exposures. In order to monitor human RF-EMF exposures and investigate potential health effects, an objective assessment of RF-EMF exposures is necessary. Accurate dosimetry is essential for any investigation of potential associations between RF-EMF exposure and health effects in human populations. This review updates state-of-the-art knowledge of currently available RF-EMF exposure assessment tools applicable in human epidemiological studies. These tools cater for assessing RF-EMF exposures in human environments; through mobile phone-based tools or other standalone tools. RF-EMF exposure assessment has been significantly improved through the application of some of these tools in recent years.

## Introduction

Radiofrequency Electromagnetic Field (RF-EMF) is typically defined as non-ionizing radiation in the frequency range of 100 kHz–300 GHz [1, 2]. Many common broadcasting and telecommunication technologies operate within this frequency range, particularly from around 1 MHz up to 6 GHz. Examples include AM radio, 526–1606.5 kHz, [2] at the lower end and Wireless Local Area Network (WLAN), 5.15–5.85 GHz, at the upper end [3]. The development of mobile telecommunication technology has evolved from its first generation (1G) Analog service (Advanced Mobile Phone Service (AMPS) to the current fifth generation (5G) service. These generations have often introduced new frequency ranges and transmission protocols. The 1G mobile phone technology operated at 800 MHz, and the second generation (2G) Global System for Mobile Communications (GSM) and Code Division Multiple Access (CDMA) operated at 850, 1900 or 900 and 1800 MHz [2]. The third (3G) Universal Mobile Telecommunications Service (UMTS) operated at 800–900 MHz range or 1700–2100 MHz; whereas the fourth generation (4G) Long Term Evolution (LTE) operates across different frequencies of 700 MHz, 1700/2100 MHz and the 2500–2690 MHz [2]. Currently the fifth generation (5G) New Radio (NR) infrastructure utilises frequencies below 6 GHz [4], but in future 5G NR will utilise frequencies in or near the millimetre wave (30–300 GHz) range [5, 6]. In Australia, the Australian Communications and Media Authority (ACMA) has made spectrum in the 26 GHz (25.1–27.5 GHz) and 28 GHz (27.5–29.5 GHz) bands available for 5G applications [7].

The allocation of a particular frequency or frequency range largely depends on national spectrum management agencies, such as the ACMA in Australia [8] and the Federal Communications Commission in the USA [9]. The development of telecommunication technologies with the potential to improve digital communication services (voice, data, video, and beyond) has led to their increasing popularity globally. According to the International Telecommunication Union, access to 4G networks and the Internet are utilised by ~ 85% [10] and over 57% [11] of the global population, respectively. More recently, 5G technologies and networks are being developed and deployed internationally [9]. The increased energy and spectrum efficiency offered by these networks permit increased capacity and speed, and the resulting possibility of plethora of new applications such as smart homes and buildings, smart cities, 3D video, work and play in the cloud, remote medical services, virtual and augmented reality, etc. [12].

In parallel with the increasing use of telecommunication and other RF-EMF emitting technologies, there are concerns in some sections of the community regarding potential adverse health effects from exposures to RF-EMF from these technologies [13]. Given that anthropogenic sources of RF-EMF exposure have increased considerably in recent decades [1], there is a need to assess these exposures. Such assessments are important to evaluate any relationship between RF-EMF exposures and potential adverse health effects in human populations.

Historically, assessment of RF-EMF exposure has been a challenging task in human epidemiological studies [14]. A major challenge has been to objectively quantify personal RF-EMF exposures to minimise exposure misclassification. Epidemiological studies have often relied on subjective and less precise methods of exposure assessment both in terms of exposure estimation and classification of participants of studies into exposed or non-exposed populations [14, 15]. For example, using ‘distance from the nearest base station’ as a measure of RF-EMF exposure [16]; or self-reported weekly number and/or duration of calls made or received on a mobile phone as a measure of mobile phone handset related personal RF-EMF exposure [17, 18].With the development and utilisation of more sophisticated RF-EMF exposure assessment tools in recent years, this challenge has been partly addressed [15, 19, 20]. Since the type of exposure assessment tool(s) and associated methodology used in human epidemiological studies affect their validity, appropriate use of RF-EMF exposure assessment tool remains an important issue.

A review on instruments to measure environmental and personal RF-EMF exposure for epidemiological studies was published in 2016 [14]. Since the review was published, telecommunication technology has evolved considerably including the introduction of 5G networks. This has subsequent ramifications for the development and application of the RF-EMF assessment tools that enable measurement of environmental and personal RF-EMF exposures. Therefore, there is a need to update the knowledge of RF-EMF exposure assessment tools, in line with recommendations for review updates on a particular topic [21].

The purpose of this review paper is to update the knowledge of state-of-the-art instruments that can be applied in objective evaluations of the RF-EMF exposures in human epidemiological studies.

## Methods

Consistent with our previous methods on this topic [14], a literature search was performed using the MEDLINE, Scopus and Google Scholar databases. The key words: “RF-EMF”, “RF-EMR”, “radiofrequency-electromagnetic radiation exposures”, “radiofrequency-electromagnetic field exposure”, "dosimeter", “personal dosimeters”, "personal measurements", "individual measurement", "exposimeter", “personal exposimeters”, “software modified phones”, “hardware modified phones”, and “apps for mobile phone exposure assessment”, were used singly or in combination. Peer reviewed articles (published in the English language) since 2015 were considered. This time frame ensures that this work includes all relevant updates on this topic since our previous review [14]. In addition, conference proceedings of the Bioelectromagnetics Society and the European BioElectromagnetics Association from the same range of years were also included. Relevant online information/publications of government agencies and the RF-EMF exposimeter manufacturers were also considered. If needed, personal contact with the manufacturers or researchers using the tools was conducted in order to update the technical specifications of their products.

Any tools/apps or exposimeters/monitors that were discussed in our previous publication [14] are not included in this paper, unless new updates were available (e.g., versions or validation findings). Only the smart-phone based tools were included in this review.

## Results

The tools used in the assessment of RF-EMF exposures in human environments are grouped into; mobile-phone based and standalone tools (e.g., exposimeters and other devices). These tools involve the measurements of RF-EMF exposures from different RF-EMF sources, their associated frequencies and technologies.

## Mobile phone-based tools

We found seven mobile phone-based apps or tools that have been used in assessing mobile phone handset related and/or Wi-Fi RF-EMF exposures. They were in the form of either apps/software, such as, XMobiSense™ [14], Quanta Monitor™ [14], ElectroSmart™ [22], Wi-Fi Radiation Meter™ [23, 24], Crowdsourcing-Based EMF Exposure Monitoring app [25, 26], QualiPoc Android™ [15] or hardware devices, such as DEVIN device [27]. Almost all (i.e., except DEVIN) were limited to mobile phones supported with Android operating systems. These tools have been used in validation studies on RF-EMF exposure assessment, as mentioned below. A research group in Japan also designed and used a Software Modified Phone [28, 29]. However, we could not find information on technical details and hence it is only briefly mentioned here. Table 1 includes updated details on types, specifications and measurement capabilities of currently available mobile phone-based instruments. The details on the application of these tools in human epidemiological studies are discussed below.

**Table 1:** Mobile phone-based RF-EMF exposure assessment instrument

Instrument name (country of manufacture)	Parameters measured	Validation or applications
XMobiSense™ (France) [14]	Date and time of incoming/outgoing voice calls, the laterality of use during voice calls, etc	Two large international studies—Mobi-Expo study [32]; COSMOS study [33]
Quanta Monitor™ (Finland) [19]	Cumulative and instantaneous parameters, including received power and transmitted power	A pilot study in Australia [19]
QualiPoc Android™ (Switzerland) [15, 34, 35]	Received Signal Strength Indicator (RSSI), Reference Signal Received Power (RSRP), and Reference Signal Received Quality	A pilot study in Australia [15]
ElectroSmart™ (France) [22]	RSSI (dB) from cellular antennas (2G, 3G, and 4G), Wi-Fi access points and Bluetooth devices, Positioning System (GPS) coordinate and the orientation of the smartphone	Two studies from France [36–38]
Wi-Fi Radiation Meter™ (Cyprus) [23]	Power density (W/m^2^ or dBm) from Wi-Fi access points	A Croatian study [24]
Crowdsourcing-Based EMF Exposure Monitoring App (Germany) [25, 26]	RSRP measurements (dB) of LTE networks, GPS coordinates, timestamps	Two studies from Germany [25, 26]
DEVIN device (France) [27]	Effective Tx power (dB), associated with cellular and Wi-Fi networks, from mobile handsets	A study from France [27]
Software Modified Phone (Japan) [28, 29]	Frequency and duration of calls; usage of hands-free kit, call types (e.g., incoming/outgoing, voice or data); transmitted and received power during a call, laterality	Two Japanese studies [28, 29]

### XMobiSense™

We have described XMobiSense™ (Whist Lab, Institut Mines-Te´le´com/Orange, Paris, France) and its technical details relevant to exposure assessment in our previous publication [14]. Following the publication, the validation data on self-reported and objectively collected measures of mobile phone usage with XMobiSense™ are available [30–32]. This app is probably the most widely used smart phone (Android)-based app that has been used in RF-EMF related epidemiological studies [32, 33].

There are several large multinational studies to use XMobiSense™ such as the Cohort Study of Mobile Phone Use and Health (COSMOS) study, a European research study, which used this app to collect data on mobile phone usage of and exposures to participants in a prospective study [33]. There is also the international prospective cohort study consortium (the UK, Sweden, The Netherlands, Finland, Denmark and France) on mobile phones and health, which includes more than 300,000 study participants aged 18 + years and will be followed up for 25 + years. Similarly, the Mobi-Expo study, also gathered proxy measure of mobile-phone related RF-EMF exposure to its participants [32]. The study involved young people (aged 10–24 years) from 12 countries worldwide; Australia, Canada, France, Germany, Greece, Israel, Italy, Japan, Korea, New Zealand, Spain, and The Netherlands [32].

There are also smaller studies using XMobiSense™. A study of 96 participants (aged 25–66 years) from the Netherlands collected RF-EMF exposure related data over 4 weeks. The data were used to compare a range of variables (e.g., number of calls, duration of calls, laterality of phone use, hands-free phone use–wired headset, Bluetooth, speaker mode) with the tool and those collected through self-reported questionnaire after 6-months [30]. Similarly, another study collected weekly data on number of calls, duration of calls (minutes) and data usage (megabytes) from 26 participants from France, Spain, and the Netherlands using the tool over 4 weeks [31]. The study compared the app data (e.g., mobile phone usage) against the data collected through self-reported questionnaire.

The application of XMobiSense™ in human epidemiological studies has improved the understanding of observed potential recall bias that research participants are likely to report on mobile phone usage. Mobile phone usage has been a conventional proxy measure to RF-EMF exposures among mobile phone users. Therefore, the application of XMobiSense™ helps to better characterise mobile phone use associated RF-EMF exposures in human populations. Such information is valuable for enhancing understanding RF-EMF exposure models based on self-reported mobile phone use [30–32].

### Quanta Monitor™

Quanta Monitor™ (Cellraid, Oulunsalo, Finland) gathers objective data on mobile phone usage and associated near-field RF-EMF exposures. Only one publication from Australia was found, that used the app to characterise mobile phone related RF-EMF exposures [19]. The study validated the tool through a pilot epidemiological study to investigate received power (Rx) and transmitted power (Tx) densities in a sample of 10 participants (aged 24–62 years) over two months. Daily objective data on their mobile phone associated RF-EMF exposures, including Tx and Rx, attributed to different modes of phone usage (e.g., cellular calls, cellular data and Wi-Fi) were collected in the study [19].

The study showed the app could be potentially employed in prospective assessment of mobile phone associated RF-EMF exposures. The exposure parameters, particularly Tx and Rx, were able to be grouped into those resulting from cellular calls, cellular data and Wi-Fi.

### QualiPoc Android™

The Qualipoc Android™ (Rohde & Schwarz, Munich, Germany) is based on commercial Android smartphones or tablets [15, 34]. The tool is supported by all mobile network technologies and has been used in radiofrequency signal optimisation and mobile telecommunication network testing, such as for troubleshooting voice, data service quality and video streaming [34].

The handset baseband chipset of the the Qualipoc Android™ gathers data on different indicators of RF-EMF signal strength, including Tx power [15, 34]. Received power exposure indicators include Received Signal Strength Indicator (RSSI), Reference Signal Received Power (RSRP), and Reference Signal Received Quality (RSRQ) for the 4G network and Received Signal Code Power (RSCP) for the 3G network. RSSI is a measure of power in a received radio signal. RSRP is a similar measure to RSSI and is the power of the LTE Reference Signals. RSRQ is the ratio of RSRP to RSSI multiplied by the number of resource blocks, its equal to (N × RSRP)/RSSI where N is the number of resource blocks used. This is a measure of the quality of the received reference signal from the mobile phone base station to the device. Simultaneously collected data on RSSI and Received Signal Code Power (RSCP) occurs when the 3G network is in use and RSSI for the 2G network. These parameters provide relevant measures of signal strength for the respective networks. RSSI, RSRP, and RSCP are recorded in dBm while RSRQ is measured in dB.

Importantly, QualiPoc records Mobile phone Tx power in dBm across all mobile phone data technologies. It collects radiofrequency signal related measurements data for a range of mobile phone technologies such as LTE—Frequency-Division Duplexing (LTE-FDD) and Time division (TD)-LTE, High Speed Downlink Packet Access, High Speed Uplink Packet Access, High Speed Downlink Packet Access DC, Wideband Code Division Multiple Access, Enhanced Data for Global Evolution (EDGE), General Packet Radio Service (GPRS), GSM, CDMA 2000®, Evolution-Data Optimized (EVDO) Rev, Wi-Fi and 5G [35]. The data recorded by the Qualipoc Android™ are stored on the handset and can be downloaded into a CSV file.

**Fig. 1:**
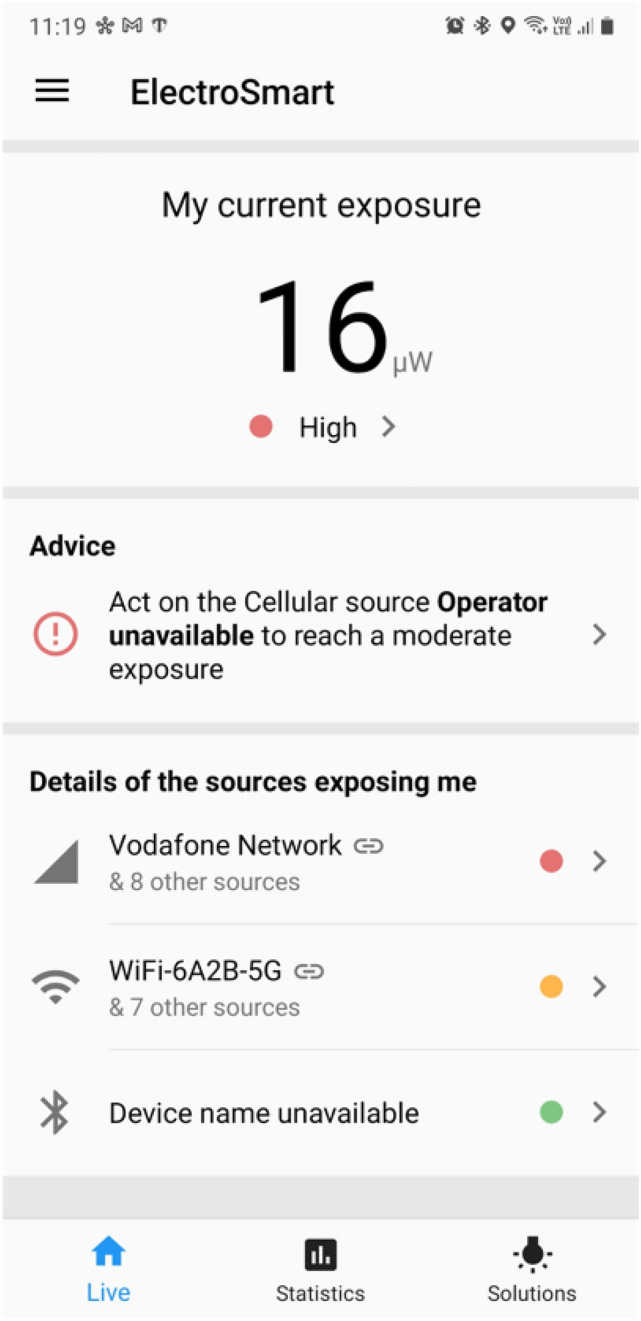
ElectroSmart™ App showing a user’s RF-EMF exposures from cellular and Wi-Fi sources (*Source* SH, ARPANSA)

A study from Australia demonstrated that the use of the Qualipoc Android™ tool in a handheld mobile phone was able to assess the correlations between multiple signal strength indicators and Tx power on the 3G and 4G networks [15]. Strong negative correlations were found between Tx and various received signal strength indicators for 3G and 4G mobile phone technologies (3G RSSI-0.93, RSCP-0.93; 4G RSSI-0.85, RSRP-0.87) indicating that large increases in Tx power occurs as the received signal level decreases [15]. The study also demonstrated strong positive correlations between RSSI and RSRP for the 4G network, and between RSSI and RSCP for the 3G network. Nevertheless, RSRQ showed only a moderate correlation between RSSI and RSRP in the 4G network [15]. The latest version of Qualipoc Android™ system is able to measure RF-EMF signal or exposure from 5G carriers [35]. Figure 1a shows a screenshot of mobile-phone based assessment data (e.g., 5G) as displayed on a Qualipoc Android™ system.

### ElectroSmart™

The French National Institute for Research in Computer Science and Automation (Inria, France) has recently developed the ElectroSmart™ app (Android-based) that provides measure of the RSSI for cellular antennas (2G, 3G, and 4G) Wi-Fi access points and Bluetooth devices [22, 36]. The RSSI (dBm) measurements are collected every 20 min in the background [37]. The app also gathers the Global Positioning System (GPS) coordinate, the orientation of the smartphone, the information on the emitting sources such as Customer Interface Device (CID), Service Set Identifier (SSID), and Basic Service Set Identifier (BSSID) when the measurement is performed [37]. Figure 1 shows a screenshot of the ElectroSmart™ app showing RF-EMF measurement data from cellular and Wi-Fi networks.

The accuracy of the RSSI measurements for LTE networks depends on the orientation of the mobile phone, the transmitting RF-EMF source, and the source positioning and orientation [38]. For mono-polarised antenna (e.g., indoor controlled environment), the calibration helps to enhance the accuracy less than 5 dBm root mean square error compared to a professional equipment [38]. The details on the evaluation and correction for the device orientation, the source position and orientation and the source Tx power are provided elsewhere [38]. The Bluetooth RSSI measurements in a controlled indoor setting and outdoor environments are sensitive to the device orientation. However, for multi-polarised antenna, such as found in LTE networks, the effect of device orientation on the RSSI is minimal [38]. The lower and upper RSSI detection limits of ElectroSmart™ app for cellular, Wi-Fi and Bluetooth are − 51 to − 113 dB, − 1 to − 126 dB, and − 1 to − 150 dB, respectively.

This app is freely available on Google Play and claims 500,000 monthly unique users worldwide since 2016. It provides the RSSI exposure data (i.e., received power) related to the above-mentioned RF-EMF sources [38]. The historical data on the RSSI for each user is stored for one month or longer in a secured server. Recently, the findings on the assessment of population (254,410 unique users in 13 countries) level RSSI exposures have been published [37]. This study showed that the ElectroSmart™ app could be a potential tool to measure RSSI exposures from various RF-EMF sources [37]. The study indicates Wi-Fi and Bluetooth contributed more than mobile phone signals to the total measured RF-EME exposures [37].

### Wi-Fi Radiation Meter™

Wi-Fi Radiation Meter™ (Sigint Solutions Ltd, Nicosia, Cyprus) is a smart phone (Android)-based app that measures RF-EMF exposure (i.e., power density or electric field intensity) from Wi-Fi access points at 2.4 GHz and 5.2 GHz [23, 24]. The app is freely available for public download (free and paid versions) via Google Play [20]. Figure 1c shows a screenshot of Wi-Fi Radiation Meter™ app showing Wi-Fi measurements.

A Croatian study used this app to evaluate Wi-Fi exposures in several human environments (e.g., homes, shopping malls, cafes, etc.) [23]. The researchers also compared accuracy of the app by comparing its data (power density) and those provided by SRM-3000 [24]. The measurement error of the app was reported to be 12% (~ 1 dB). The study concluded that the Wi-Fi Radiation Meter™ has a potential to be used as a tool for assessing Wi-Fi exposures in human environments.

### Crowdsourcing-based EMF exposure monitoring app

The scientists from RWTH Aachen University and Fraunhofer Institute for High Frequency Physics and Radar Techniques, Germany have recently developed and tested this app. This Android app measures the transmitted signals from LTE networks station (i.e., RSRP) [25, 26]. In addition, it also records RSSI, RSRQ, location information (GPS coordinate) and a time stamp; all data are logged every half or full second. Using the app, the RSRP measurements associated with the German LTE networks (i.e., 800 MHz, 1800 MHz, 2100 MHz, and 2600 MHz) were conducted [25, 26]. The RSRP data were then converted into field strength values by using previously determined conversion factors for the individual smartphones [26]. The Kriging method is used to smooth the noisy measurement data (converted into field strength values) of the smartphones and to perform an interpolation [25]. In this process, the data of each radio cell is treated individually, and the results of the individual cells are then combined to calculate the total exposure of the LTE network. The derived exposure values were compared with field strength measurements carried out with SRM-3006 (code-selective mode). It was observed that a relatively stable relationship between predicted RSRP (interpolation) and electric field strength measured (SRM-3006) was obtained for different locations with several smartphone measurements. The difference between the E-field strength and the interpolated RSRP values ranged from 10 to 24 dB [25]. Details on these steps and measurements are available elsewhere [25]. Further validation studies using this app will help determine its usefulness in view of its application in RF-EMF epidemiological studies. However, the available data [25, 26] support the claim that the RF-EMF exposure data collected by the app would be useful in assessing RF-EMF exposures from LTE networks.

### DEVIN device

A group of scientists from France have recently developed and tested DEVIN device, a mobile phone attachable tool [27]. This device can be attached to the user’s smartphone or tablet to measure the effective Tx power (dB) associated with cellular and Wi-Fi networks (accuracy ± 1.5 dB) [27]. Typically, it records root mean square (RMS), maximum and average Tx powers values that are saved on a SD card automatically while the device is in use. The sampling frequency ranged from 1 Hz and 1 kHz [27]. DEVIN has been calibrated (in free space) for six uplink frequencies of cellular bands and two Wi-Fi bands [personal communication, Serge Bories, the French Atomic Energy Commission]. Details on calibration of four frequency bands (i.e., two cellular frequency bands of 1747 MHz and 847 MHz; and two Wi-Fi bands at 2.4 GHz and 5.2–5.7 GHz) have been published [27]. The raw data can later be transferred to a computer through a USB link, the data are analysed by using calibration table offline [36]. DEVIN must be calibrated for each mobile phone/tablet model and frequency band before the device is used for dosimetry [27]. Further development is underway to include its capability to measure 3.5 GHz and Wi-Fi 6 GHz frequencies. The tool will be used in a French epidemiological study involving over 300 volunteers [27]. The findings of this study will be valuable to assess its practical application in in RF-EMF epidemiological studies.

## Standalone tools

These tools include exposimeters and other RF-EMF exposure or radiofrequency spectrum monitoring tools (e.g., spectrum analyzer) which can either be used for environmental and/or personal RF-EMF exposure monitoring. Environmental monitoring consists of undertaking a static spot measurement or dynamic drive through measurements. Personal RF-EMF exposure monitoring is used for assessing individual exposures to members of the general public, including at occupational settings. The standalone tools included here are ExpoM-RF 4™ (Fields at Work, Zürich, Switzerland), EME Evolution™ (Satimo, Cortaboeuf, France), Personal distributed exposimeter (PDE) or Whole Body Worn Exposimeter (WBWE), PDE-Helmet and Drone-based RF-EMF measurement nodes (Ghent University/iMinds, Ghent, Belgium), SRM-3006™, NBM-550™, RadMan 2XT™ and RadMan 2LT™ (Narda Safety Test Solutions GmbH, Pfullingen, Germany), and RFeye™ systems (Chantilly, VA, USA). Though SRM-3006™ and NBM-550™ stand out as different to all the other body-worn exposimeters, we have summarised them here as they have been used in assessing environmental RF-EMF exposure levels. The applications of these instruments in human RF-EMF exposure assessments are briefly summarised here. Further, Table 2 below summarises the characteristics, such as frequency range, exposure measure type and sensitivity of these tools.

**Table 2:** Stand-alone tools for environmental and personal RF-EMF assessment

Type/name (country of manufacture)	Frequency bands (range)	Sampling intervals (s); data storage capacity/data points	Detection limits (measurement range)	Parameters measured	Size (L × W × H, cm); Weights (g)	Battery lives	GPS (Global Positioning System)/Geolocation function	Measurement uncertainties (vendors’ data)^a^	Validation and applications
ExpoM-RF 4™ (Switzerland) [39]	25 frequency bands (50 MHz–6 GHz)	3–6000 s; 16 GB	5 mV/m–6 V/m (high sensitivity mode); up to 60 V/m (high field strength mode)	RMS and max E-field strengths (V/m)	16 × 8 × 4; 360	Variable (e.g., > 15 h with 25 bands, 5 s interval and GPS off)	Available	4.5 dB (decibel) (expanded uncertainty) [in free space]	Environmental/personal exposure assessments, akin to ExpoM-RF 3™ [40, 41]
EME Evolution™ (France) [42]	20 frequency bands (8 MHz–6 GHz)	2–255 s; NA	0.02–6 V/m	RMS, min and max E-field strengths (V/m)	17.6 × 7.3 × 4.8; 520	~ 1–7 days depending upon measurement scenarios	Available	NA	Environmental/ personal exposure assessment
Exposimeter [43] (Spain)	(78 MHz–6 GHz)	resolution bandwidth 300 kHz; sweeping time ~ 902 ms; SD card memory up to 2 GB	− 70 to + 20 dBm (decibels per milliwatt)	max E-field power density (in dBm; 0 dBm = 1 mW), which needs to be converted into the power density (in W/m^2^) [43]	8.5 × 7.0 × 2.2; 250	–	Available	0.04 dB [in free space]	Environmental/personal exposure assessment [43]
Personal distributed exposimeter (PDE) or Whole Body Worn Exposimeter (WBWE) [20, 44, 45]	Up to 11 frequency bands: 4 frequency bands (900 DL and UL, MHz, Wi-Fi 2.4 GHz, 2.6 GHz) [15]; 10 frequency bands (800, 900 UL/DL, 1800 UL/DL, 2100 UL/DL, 2600 MHz, DECT 1900 MHz, Wi-Fi 2.4 GHz); 11 frequency bands (790 MHz-5.5 GHz) [39]	1 s or extendable (2–90 s)	6 mV/m (lower detection limit)	average power density (W/m^2^)	Multiple receiving antennas, attached to a vest	–	NA	3.4–5.5 dB [on-body]	Personal exposure assessment [20, 44, 45]
PDE-Helmet [46]	925–960 MHz	1 s	11.3 mV/m–113 V/m (on-body calibration)	average power density (W/m^2^)	4 stub antennas (aperture = 1.8 cm^2^) embedded in a helmet	Up to 48 h [personal comm. Arno Thielens, 16/6/2021]	NA	5.5 dB [on-body]	Head-specific exposure assessment [46]
SRM-3006™ (Germany) with multiple probes [47–51]	9 kHz–6 GHz	User selectable	0.2 mV/m–200 V/m (27 MHz–3 GHz); 14 mV/m–160 V/m (420 MHz–6 GHz) [E-field probes]	average, min and max E-field strengths (data and spectrum modes)	21.3 × 29.7 × 7.7; 2800 (excl. cable connectable probes)	2.5 h (with GPS)	Available	Frequency specific (e.g., + 3.3/ − 5.3 dB for 2700–3000 MHz; + 3.1/ − 4.9 dB for 5000–6000 MHz) [in free space]	Environmental exposure assessment [47–51]
NBM-550 ™ (Germany) with multiple probes [52]	100 kHz–50 GHz (E-field); 300 kHz–30 MHz (H-field) [53]	5 s (manual measurement); 5, 50, 60 s (remote control measurement)	Probe-specific (EF 0691, 0.35–650 V/m [100 kHz–6 GHz] [E-field]); HF 0191 0.018–16 A/m 27 MHz–1 GHz [B-field]	average, min and max E-field strength (V/m)/B-field strength (A/m)			Available, if added as an additional module	± 1.5 dB (1 MHz − 4 GHz) [53] [in free space]	Environmental exposure assessments (occupational/the general public) [53]
RFeye™ systems (USA) [54]	9 kHz–8 GHz, 9 kHz–18 GHz, or 9 kHz–40 GHz	NA	NA	NA	NA	NA	NA	NA	Exposure monitoring over a geographic area [54, 55]
RadMan 2XT ™ [56] RadMan 2LT ™ [56]	50 MHz–8 GHz (E-field); 50 MHz–1 GHz (H-field) [LT] 900 kHz–60 GHz (E-field); 27 MHz–1 GHz (H-field) [XT, ICNIRP 98 Occ models]	1 s (XT), 1 s–30 ms (LT, pulse mode) [integration time] 2880 events (1-min logging interval) [LT] 100,000 events (1–6 min logging interval) [XT]	< 1% of standard (RMS E-field and B-field strengths)	5%, 10%, 25%,50%, 100%, and 200% of the standards; vibration and audible sound activation at 50% and 100% of the standard	165 × 47 × 31 mm; 185 g	800 h of operation;	NA	± 3.5 dB (LT); frequency dependent for XT, e.g., ± 3 dB (900 MHz–0 GHz) [E-Field] ± 3 dB (LT); ± 3 dB (XT, 27 MHz-1 GHz) [H-Field] [in free space]	Personal exposure assessment (occupational/the general public); % of the standard means any national or international RF-EMF exposure limits, including ICNIRP
Drone-based RF-EMF measurement nodes [57]	3-D RF-EMF exposure assessment up to 60 m height above ground (900 MHz)	1 s		RMS electric field strength (V/m) or average power density (W/m^2^)	–	15–20 min	NA	2.3 dB [in free space]	3-D environmental RF-EMF exposure assessment [57]

*NA* not available, *GB* gigabyte, *DL* downlink, *UL* uplink, *ICNIRP *International Commission on Non-Ionizing Radiation Protection, *SD* Secure Digital, *dB* is a standard unit used to measure the intensity of the power level of an electrical not available signal by comparing it with a given level on a scale, *GPS* global positioning satellite, *3D* 3-dimensional

^a^Measurement Uncertainties: for PDE,/WBWE and PDE-Helmet it is ob-body calibration, for the others it is in free space. Note: parameters measured at frequencies exceeding 1 GHz are E-field only

**Fig. 2:**
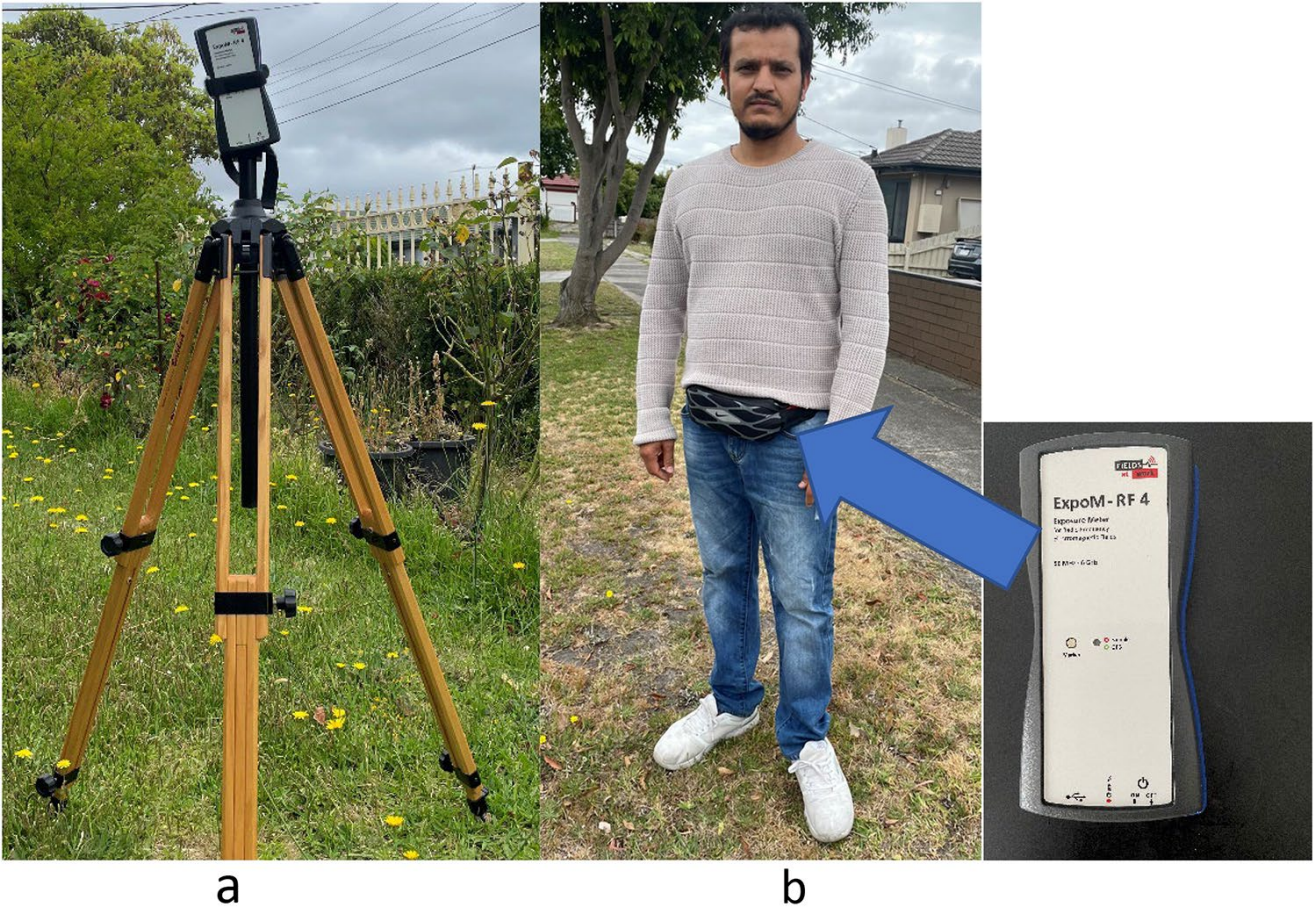
ExpoM-RF 4™ exposimeter in a spot (**a**) and personal (**b**) RF-EMF exposure measurements [*Source* CRB, ARPANSA]

Exposimeters have been used in environmental and personal RF-EMF assessment studies. The most commonly used exposimeters include, ExpoM-RF series, ExpoM-RF3™ [41, 46, 58–63] and the EME Spy series (Satimo, Cortaboeuf, France) [62, 64, 65]. These tools have been used to undertake various types of RF-EMF measurements, such as spot measurements, microenvironmental and personal assessments in different contexts. The most recent versions of these tools (e.g., ExpoM-RF4™ and EME Spy Evolution™) have only been marketed in the past few years. Figure 2 shows ExpoM-RF4™ being used in a spot and personal RF-EMF exposure assessment.

Researchers from Belgium have developed and tested a personal distributed exposimeter (PDE) or body-worn distributed exposure meter (BWDM) with multiple textile antennas that can be fitted into a garment [20, 44, 45]. In addition, they have also developed a helmet-fitted exposimeter with antennas attached into a helmet that was specially designed to measure RF-EMF exposure to the head [46]. However, the PDE/BWDM and the helmet-fitted exposimeters are not yet commercially available and were only used for pilot or validation studies [20, 46]. A recent study [44] describes personal RF-EMF exposure measurement surveys in five European countries.

An exposimeter device, also capable of functioning as a spectrum analyser, has been recently designed and tested in Spain [43]. The device claims to be capable of sampling 20,000 samples per second, taking less than one second to measure the frequency spectrum of 78 MHz–6 GHz. Its dynamic power (W/m^2^) measurement range is of 90 dB with an input power ranging from − 70 to + 20 dBm. With a 0.04 dBm resolution, the system measures or detects all the RF-EMF exposures in multiple narrow bands of 300 kHz.

**Fig. 3:**
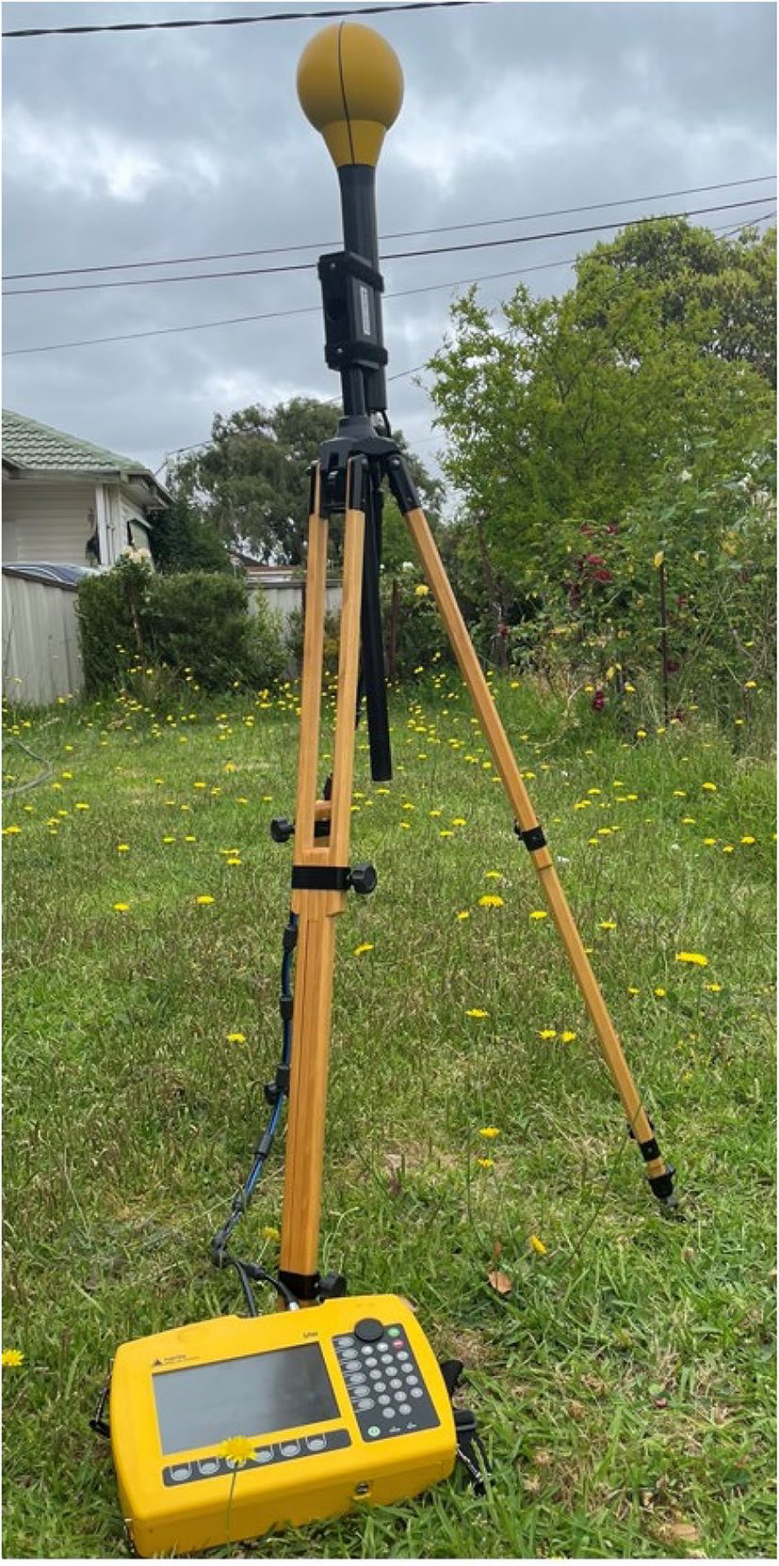
SRM-3006.™ spectrum analyser used in a spot RF-EMF exposure assessment [*Source* CRB, ARPANSA]

This tool, still under development, was used in conducting RF-EMF spot measurements in the frequency range of 791–2170 MHz across seven residential locations in Madrid. The measurements (maximum power density levels) were compared to the spot measurement data collected with the FSH8 portable spectrum analyzer (connected to a TSEMF-B2 omnidirectional antenna) (Rohde & Schwarz, Munich, Germany) [43]. This validation study demonstrated that RF-EMF exposure levels reported by these two tools were similar (Pearson’s correlation coefficient = 0.98). Similarly, other tools have wide applications in environmental RF-EMF assessments. For example, the Selective Radiation Meters, viz*.* SRM-3000™ and SRM-3006™ (Narda Safety Test Solutions GmbH, Pfullingen, Germany) have been widely used in RF-EMF exposure assessments [47–51]. These handheld spectrum analysers are capable of monitoring a number of frequency bands for RF-EMF exposure assessment from a variety of technologies. They have been mainly used for undertaking spot or environmental RF-EMF assessments to measure exposure across all frequency ranges [47] or a specific frequency [49]. Table 2 includes SRM-3006™, which is the latest version of the series. Figure 3 shows SRM-3006™ being used in a spot RF-EMF exposure assessment. A number of other Narda products are also available to undertake RF-EMF measurements, including NBM-550™, which is a broadband probe and detector [52].

Another broad-band monitoring system that was used as a car-mounted mobile measurement system is the RFeye node™ [55]. It can be used in a static/fixed site or mobile system (e.g., car/van) to monitor RF-EMF exposures from several RF-EMF emitting technologies, including telecommunication systems. Publications by researchers in the UK and the Netherlands describe the use of CRFS Eye™ with NOD-I 0001 (one of the past models of RFeye node™) system attached to a car-roof to assess mobile phone base station exposures (900 MHz and 1800 MHz) over a large area [55]. This driving-mode RF-EMF monitoring system claimed that it provides reliable RF-EMF measurement data which are comparable to those given by exposure modelling or the body worn exposimeter. Newer versions of RFeye node systems are available [54] and included in Table 2.

There are occupational RF-EMF exposure assessment tools (RadMan radiation monitors) [66–68] that have been mainly used for monitoring occupational RF-EMF exposures that involves anticipated high RF-EMF exposures compared to the general public environments. For instance, two studies report on occupational RF-EMF exposure monitoring in military and medical settings [67, 68].

Several other types of RF-EMF monitoring systems have been reported and are on the market. However, we have not included them into this paper due to their limited applications as noted in the scientific literature. Table 2 only includes the latest versions of the tools we reviewed in our previous publication [14] or the others radiation meters (e.g., SRM models) that have been described in the literature elsewhere [47–51].

For environmental RF-EMF assessments, drone-based or drone supported measurement systems have been developed and tested. One of such tools was designed and tested by a research group in Belgium [57]. This was a prototype of a drone-based RF-EMF measurement tool involving measurement of 900 MHz RF-EMF exposure from base stations. It had three identical nodes and associated electronics, and three orthogonal lightweight monopoles or alternatively three linearly polarized planar patch calibrated antennas. This triaxial hexacopter drone system with an isotropic antenna, allowed collection of RF-EMF exposure data at various altitudes ranging from3 to 60 m [57]. Figure 2a shows a take-off picture of a drone-based measurement node in action.

Similarly, researchers from Hungary and Greece piloted a drone-supported monitoring system that attached commercially available exposimeter or RF-EMF monitor (e.g., ExpoM-RF, EME-Spy or SRM-3006) to a robust drone [69–71]. As shown in Fig. 2b, the Hungarian measurements have involved attaching exposimeters/meters in different configurations of the drone system [70, 71]. The Hungarian group has recently tested feasibility of measuring 5G NR (3.6 GHz) exposure attaching ExpoM-RF™ and SRM-3006™ on the bottom side of a drone [71]. These studies have demonstrated drones as a promising integrative tool to undertake broadband and band-selective RF-EMF measurements at different altitude/space that may be challenging to undertake otherwise.

## Discussion

This paper provides an update on the currently available near-field and far-field RF-EMF exposure assessment tools for human RF-EMF exposure assessment. Some of the tools discussed here are new, while others have been previously reviewed [14]. In addition to presenting the new tools, we have included the findings on the validation studies of the previously reviewed tools. This paper discusses the main technical specifications of the tools, identifying their strengths and limitations for use in RF-EMF exposure assessment studies.

## Mobile phone-based tools

Several reports of phone-based RF-EMF exposure tools used in epidemiological studies, including smart phone-based apps or tools, have been published in the past decade. In particular, XMobiSense™, Quanta Monitor™, QualiPoc Android™, and Software Modified Phone (SMP) have been applied to objectively assess a number of RF-EMF exposure parameters associated with mobile phone usage [19, 25–27].

Although XMobiSense™ measures Rx power on the installed handset, such data are not available in the literature. Only limited published research has demonstrated the application of Rx and Tx power as a mobile phone associated RF-EMF exposure measure [15, 19]. Though SMP apparently report on Rx and Tx, details about their measurements are unavailable [28, 29]. Two pilot studies [15, 19], involving Quanta Monitor™ and the QualiPoc Android™, have described the characterisation of Rx and Tx exposure parameters in relation to the use of a mobile phone by human subjects. The studies demonstrated that the collection of data on Rx and Tx powers (in addition to number and duration of calls) can be a promising measure of RF-EMF exposures to improve mobile-phone related RF-EMF exposure assessment in future epidemiological studies [15, 19].

Of seven mobile-phone based tools that are discussed here, only four (ElectroSmart™, Crowdsourcing-Based EMF Exposure Monitoring App, DEVIN exposimeter, Wi-Fi Radiation Meter™) are new tools reviewed since our last publication [14]. The limitations of these tools are that none of them (except DEVIN device) are able to assess RF-EMF exposures emanating from iPhones/iPads or non-Android based devices. Clearly, this may introduce selection bias while using these tools in human epidemiological studies unless iPhone and non-iPhone users have similar usage patterns. The DEVIN device is able to measure Tx power from any device; while the others only assess Rx power from cellular, Wi-Fi and Bluetooth devices. Further, except for Qualipoc Android™ system, other mobile phone-based tools apparently are unable to measure 5G associated RF-EMF exposures. To-date mobile phone-based tools have only been used in a few studies with small sample sizes [15, 19]. Further validation studies involving a large number of human subjects are needed to collect more reliable data on RF-EMF exposures from mobile phone or other near-field device (e.g., tablet) usage. ElectroSmart™ was used in collecting RF-EMF exposure data of its global users consisting of a large population [37]. A recent study claimed that RF-EMF exposures to Wi-Fi, Bluetooth and cellular base stations are increasing; and of them, Wi-Fi and Bluetooth sources contributes to about 50–90% of the total measured RF-EMF exposures [37]. However, the reported RF-EMF exposures can only be a surrogate measure of population exposure unless the mobile phones (with the App) were placed close to the body during the whole time when RF-EMF exposures were measured. ElectroSmart™, Quanta Monitor™ and Wi-Fi Radiation Meter™ are the apps that are publicly available for download via Google Play. This may facilitate using these apps for RF-EMF exposure assessments at the population scale. However, they also warn the users about their exposure level (e.g., low, moderate, high) that may alter users’ behaviours in terms of RF-EMF exposure they encounter in their daily lives. This may, in turn, provide a different exposure data compared to those if the users were not aware of their exposure levels, and therefore needs to be interpreted accordingly. A key limitation of the DEVIN tool is that it needs to be calibrated (i.e., in free space) for the user’s mobile phone to achieve reliable results. This is challenging as calibration for several mobile phone model users may not be practically possible in RF-EMF epidemiological studies. However, application of a non-specific calibration (e.g., mean from several models) measure may be possible for a user’s mobile phone model even though this may lead to some measurement uncertainties.

Since RF-EMF exposure as a result of mobile phone usage is a major part of the total RF-EMF exposure (brain and whole body) to humans [72], these tools can contribute to characterise exposure-related to mobile phones, tablets/iPads, or other near-body RF-EMF emitting telecommunication technologies. A major challenge for mobile phone-based apps has been to quantify Tx power from mobile phone handsets, which is much larger than Rx from mobile phone base station/Wi-Fi routers [20]. It is therefore be expected that the development and deployment of the DEVIN device should be able to address this challenge.

## Standalone tools

The RF-EMF standalone tools (e.g., exposimeters, exposure meters or monitors), which assess RF-EMF exposure levels in human environments have been widely used in recent years [40, 41, 68, 69]. Of the standalone tools, only the SRM-3006™, NBM-550™, and RFeye™ systems include the measurement capabilities of undertaking environmental RF-EMF exposure assessment for AM radio band. Despite the declining popularity of AM radio worldwide [73], it is still important to monitor existing RF-EMF exposure to AM radio signals as they contribute a major share to total RF-EMF exposures in outdoor environments [47]. Portable exposimeters measure frequency-band specific emissions from FM radio, TV, base stations (downlink) and mobile phones (uplink), cordless phones, and Wi-Fi [14]; and have been in use since 2005 [74]. RF-EMF exposure monitoring systems for monitoring occupational RF-EMF exposures have been in use mainly to warn RF-EMF personnel about exceedance of the regulatory limits by incorporating a preset value with an alarm/vibration and most of them do not log the measurements [74].

Several studies employed exposimeters or other tools to assess everyday RF-EMF exposure levels in different contexts mainly by utilising five main methods of exposure assessment [40, 41, 75]. They include;

(i) spot measurement performed with portable devices that can be set up temporarily at various locations (e.g., SRM-3006™, ExpoM-RF™),
(ii) personal exposure assessment with volunteers carrying a device (e.g., ExpoM-RF™ or EME Spy™) during their daily activities,
(iii) mobile microenvironmental measurements with trained researchers walking, bicycling or driving through various microenvironments carrying a personal measurement device (e.g., ExpoM-RF™ or EME Spy™) [40, 41, 75],
(iv) large area outdoor environment monitoring by driving a car covering large distances with a spectrum monitoring device (e.g., RFeye node™) on its roof [55], and environmental monitoring with a drone-based RF-EMF measurement nodes [57].
(v) Occupational personal RF-EMF exposure monitoring by attaching personal radiation monitoring devices (e.g., RadMan 2XT™ and RadMan 2LT™) usually placed in the pocket or attached to a belt, helmet or clothing [67, 68].

We can therefore characterise the studies using these tools as measurements of a specific place (spot measurement), person (personal measurement), an environment of public interest (microenvironmental measurement) [75] and a relatively large environment (area monitoring) [55]. Of note, personal radiation monitors are also able to monitor RF-EMF exposures from the RF-EMF sources that are in the close to human body while working near radiofrequency antenna.

Irrespective of the study methodology (spot, microenvironmental, personal or area monitoring) in epidemiological or ecological studies, it is obvious that these tools have an array of applications in RF-EMF dosimetry relevant to the general public, regulators and occupational populations (Table 2). The exposure metric that is mainly used to characterise RF-EMF exposure has been electric field intensity (V/m) or power flux density (W/m^2^) and most of these devices log these data while undertaking measurements (Table 2). Generally, measurement of either electric field (E-field) or magnetic field strength (H-field) intensity is sufficient while undertaking environmental (e.g., spot) or personal RF-EMF exposures [76]. Generally, E-field strength values (average/RMS, min or max) are physically measured, whereas H-field strength (RMS or average, such as S parameter) are estimated from the E-field strength values using the equations relevant to the far-field plane wave exposure scenario (i.e., $S=EH=\frac{{E}^{2}}{377}$ [77]. In the far-field exposure region, the E-field and the H-field vectors and the direction of propagation are mutually perpendicular, whereas this is not true for the near-field exposure region [77]. The RF-EMF exposures in the near field scenarios are much more complex and hence both E and H fields must be measured as electric field strength or power density alone is not an appropriate measure of near-field RF-EMF source exposure [77]. For the case of personal radiation monitors in occupational settings, where both near and far-field exposure may be encountered, a body-attached exposimeter should record both E-field and H-field exposures.

Spectrum analyser mode available in the sophisticated device such as SRM-3006™ is a useful tool to identify RF-EMF source and associated exposure levels. Recently, development of exposimeters (e.g., the ExpoM-RF4™ and the Spanish exposimeter) have considered this functionality. Exposimeters are characterised by different size, weight, number/type of frequency bands they involve, measurement interval, internal memory capacity, lower and upper detection limit, and availability of built-in GPS-logger [14, 74 see Table 2]. The availability of in-built GPS is a useful function in RF-EMF measurements (e.g., spot or microenvironmental measurements) where a reference of geolocation is needed. For example, GPS co-ordinates (available in SRM-3006™), and the GPS visualisation data, such as KML (Keyhole Markup Language) file format in Google Earth (available in ExpoM-RF4™) can be used to display/confirm geographic coordinates (in spot measurements) and navigation paths of RF-EMF assessments (area survey or microenvironmental personal RF-EMF assessment). These capabilities help both in conducting fresh and repeated measurements (e.g., RF-EMF exposure monitoring over time) with improved spatial resolution. Exposimeters, unlike spectrum analysers (e.g., SRM-3006), are compact and light enough to allow them to be worn on the body as personal dosimetry. These tools have different hardware designs (e.g., internal antenna configuration and logarithmic or RMS detector), and hence their sensitivities, associated measurement biases and uncertainties also differ [74]. Although the first microenvironmental or epidemiological studies conducted with previous EME Spy detectors used a logarithmic (log) detector, more recent models use true root-mean-square (RMS) measure [69]. In fact, the modulation and multiplexing techniques associated with today’s telecommunication technologies involve complex signals with time-varying amplitude (and phase) envelopes. The variation in the signals is measured with the peak-to-average envelope power ratio (PAR). In the log detector, the detected output varies logarithmically with the input signal PAR; whereas it is independent of the input signal PAR in the RMS detector [78]. Some weak RF-EMF signals in the environment, with field strengths below the lower detection limit of an exposimeter, are not registered by the exposimeters [60, 74] which can lead to bias in the observed measurements. In recent years, this limitation has been addressed through the application of different statistical approaches while estimating mean/median RF-EMF levels [60, 74]. Detailed descriptions on recent exposimeters, and their source of bias and uncertainties have been discussed elsewhere [74].

As shown in Table 2, narrow-band exposimeters can only measure in specific ranges of the RF-EMF electromagnetic spectrum. Therefore, they are not suitable for measuring the entire frequency range of broadcasting and telecommunication technology. This would obviously result in only a partial and limited assessment of RF-EMF exposure [74]. However, SRM-3006™ is able to measure much wider frequency ranges but are not suitable for personal exposure assessment as they cannot be worn on the body. Similarly, cross-talk is another limitation of some exposimeters (e.g., ExpoM-RF and EME Spy models) whereby RF-EMF exposures in one field strength gets measured in other frequency bands—for instance mobile phone uplink exposure may get registered into downlink bands, or a cordless phone signal may get registered into 1800 MHz mobile phone frequency band [74]. Evaluation of cross-talk for the previous ExpoM-RF3™ model has been conducted elsewhere [60]. For the latest version of ExpoM-RF4™, the expected cross-talk has been suggested in the range of − 40 dB and − 60 dB. This also depends on instrument settings, for example how wide and how far apart (in frequency) the monitoring bands are set. Similarly, only negligible systematic differences between ExpoM-RF™ devices while evaluating downlink, uplink and total RF-EMF exposures indicates that the devices offer validity in terms of RF-EMF measurements [79]. We have not been able to find similar comparisons for other exposimeters in the literature. EME Evolution™ is apparently the latest version of the previously used EME Spy models [75], which were widely used for personal measurements with volunteers, microenvironmental measurements with trained researchers and personal RF-EMF measurements with volunteers [75]. Except for the information available from its vendor [42], limited information on its application in RF-EMF assessment is available in the literature [80]. We found a study [80] that employed this particular tool in evaluating spatial characterisation of RF-EMF exposures (2G to 5G) within trams in Spain.

Personal RF-EMF measurements conducted with most of the narrow band exposimeters, when placed close to the body, may provide inaccurate exposure data (i.e., lower or higher than actual exposures) due to the physical influence of the human body and the effect of the physical environment on the RF-EMF beam [81]. A recent study that reported exposures measured with two ExpoM-RFs™, attached to the lateral sides of the hip, found estimated median exposures with the two devices were nearly 2 to 3 times higher than those measured with a single ExpoM-RF™ [60]. These results indicate the magnitude of under-estimation of exposure with a single exposimeter, due to the human body shielding effect [60]. To overcome the body shielding effect on personal RF-EMF exposure assessments, PDE or BWDM have been developed and tested to conduct measurements in a laboratory and real human environments [20, 45, 46]. The sensitivity of BWDM (800 MHz to Wi-Fi 2.4 GHz) is comparable to those of ExpoM-RF™ and EME Spy 200™ exposimeters [45]. The measurements conducted with the BWDM provided somewhat higher personal RF-EMF exposures compared to those provided by one or two exposimeters attached to the torso [45]. Furthermore, RF-EMF personal exposures (i.e., actual incident power densities) measured with ExpoM-RF™ or EME Spy™ are 1.6 to 20.6 times lower than those measured with BWDM [82]. Though PDE or BWDM have demonstrated that they provide different estimates of personal RF-EMF exposures, currently they are impractical tools for assessing exposures in the general population as they are bulky, inflexible and need to be calibrated for each person wearing the device [44]. However, personal RF-EMF exposure data gathered by BWDMs are important to interpret personal RF-EMF exposure levels in epidemiological studies [45].

Recently, Spanish scientists have published details of an exposimeter tool which comes with a spectrum analyser function [43]. However, it only provides measurement of the maximum RF-EMF levels; therefore, this tool could not be used where RF-EMF exposure assessment needs to be reported in RMS values as often reported by other exposimeters (e.g., ExpoM-RF or EME spy series). Limited data on spot RF-EMF measurements [43] and no data on personal measurements collected using this device indicates that further validation studies are needed prior to application in RF-EMF epidemiological studies.

Other RF-EMF monitoring systems, in particular RadMan 2LT™ or RFeye node™ have the advantage that they cover much wider frequency ranges. The former is used as a personal warning device for RF-EMF occupational exposures to E-and H-fields radiated by broadcast transmitters, mobile phone base stations and radar systems. The vehicle-mounted spectrum monitoring system (e. g., the RFeye node™) have been applied in RF-EMF exposure assessment of across a geographic area. This tool has a limitation that its antenna is anisotropic due to either vehicle reflection or shielding from the vehicle [55]. The issue of shielding is also obvious for human body-worn exposimeters while undertaking microenvironmental RF-EMF assessments [60, 74].

Drone-based and/or drone-attached RF-EMF exposure assessment systems have been designed or assembled in recent years. One of the benefits of these systems is that they can be used to provide 3-dimensional (3-D) RF-EMF exposure mapping of an area across different altitudes. However, the measurement program is limited by maximum airtime of the drone, drone battery capacity, wind conditions and the need of a trained pilot to fly the drone. For example, a light drone-based measurement system that was used by researchers in Belgium was limited with a flight time of 15–20 min [57]. However, airtime with more sophisticated and larger drone-based measurement systems could be longer as indicated by the drone-based measurement system being piloted by researchers in Hungary [69]. Drone-based RF-EMF measurement systems could be useful in undertaking altitude-based assessments [69–71], in particular in the locations that are generally inaccessible to humans including near to antenna, close to high-rise buildings. The measurements in such locations could provide some basis to estimate anticipated RF-EMF exposure levels, such as, close to base station installations and/or near to windows for people living in high rise buildings.

### Knowledge gaps and implications

RF-EMF exposure assessment approaches should ideally be able to integrate organ specific (e.g., brain) and whole-body RF-EMF exposures so that they provide a better estimate of total personal exposure [72]. Some progress has been made [72], but there is further scope to integrate both types of exposures in a single or multiple measurements. Similarly, accurate techniques for estimating users’ personal RF-EMF exposures from their wireless devices would be of benefit to future epidemiological studies.

Standalone RF-EMF exposure assessment tools, including exposimeters, have demonstrated their capabilities to assess exposures up to 6 GHz. It is likely that measurement approaches for 5G NR may differ slightly from conventionally applied methods, mainly because of the beam forming technology [83]. A methodology for assessing environmental or personal 5G (sub 6 GHz)-related RF-EMF has recently been proposed [83]. Furthermore, with upcoming mmWave frequency telecommunication technology, it is still not clear if and how currently available devices would allow measurements of mmWave RF-EMF frequencies. Except for the personal radiation monitor, RadMan 2LT™, which we have discussed here, none of the tools are able to measure at frequencies relevant for mmWave exposures. Therefore, it would be useful to have exposimeters radiation meters, such as ExpoM-RF and EME-models or SRM-models, which could go beyond its current upper range of 6 GHz. This is also relevant for body-distributed exposimeters. There is some ongoing development happening to address this issue. For example, SRM device, with its 5G down-converter antenna (24.5–30 GHz), is being currently designed with an aim to enable the device to conduct assessment of RF-EMF exposures from millimetre waves [personal communication, Rachit Sahay, Air-Met Scientific]. Therefore, it is likely that currently available tools will continue to evolve in terms of expanding their measurement capabilities. RF-EMF measurements performed in the close vicinity of other sources of electromagnetic fields (e.g., near a high voltage electricity supply) may show some influence of such sources. For example, a particular measurement system could be out-of-band sensitive due to the influence of such background electromagnetic fields that are not to be intentionally measured. Therefore, it should be reported by the manufacturers of the measurement system, if it may happen, or experimentally verified, if possible.

## Conclusion

This updated review includes most currently used tools for environmental and personal RF-EMF assessment. These tools, involving both mobile phone-based and standalone RF-EMF exposure assessment instruments, provide useful objective measurements of RF-EMF exposures associated with broadcast and telecommunication technologies. Most of these instruments have been validated through recent epidemiological studies conducted internationally. These tools have demonstrated capabilities in providing RF-EMF exposure data for current and future human epidemiological studies. The future research and development in the science of RF-EMF exposure assessment could consider tools that would enable exposure assessment in relation to a wide range of currently available RF-EMF emitting sources, such as cordless and mobile phones, tablet devices and laptop computers. The need for further development of exposimeters or radiation meters that could measure mmWave frequencies is also necessary.
